# Dynamic Volume Changes in Astrocytes Are an Intrinsic Phenomenon Mediated by Bicarbonate Ion Flux

**DOI:** 10.1371/journal.pone.0051124

**Published:** 2012-11-30

**Authors:** Clare M. Florence, Landon D. Baillie, Sean J. Mulligan

**Affiliations:** Department of Physiology, University of Saskatchewan, Saskatoon, Saskatchewan, Canada; Albany Medical College, United States of America

## Abstract

Astrocytes, the major type of non-neuronal cells in the brain, play an important functional role in extracellular potassium ([K^+^]_o_) and pH homeostasis. Pathological brain states that result in [K^+^]_o_ and pH dysregulation have been shown to cause astrocyte swelling. However, whether astrocyte volume changes occur under physiological conditions is not known. In this study we used two-photon imaging to visualize real-time astrocyte volume changes in the stratum radiatum of the hippocampus CA1 region. Astrocytes were observed to swell by 19.0±0.9% in response to a small physiological increase in the concentration of [K^+^]_o_ (3 mM). Astrocyte swelling was mediated by the influx of bicarbonate (HCO_3−_) ions as swelling was significantly decreased when the influx of HCO_3−_ was reduced. We found: 1) in HCO_3−_ free extracellular solution astrocytes swelled by 5.4±0.7%, 2) when the activity of the sodium-bicarbonate cotransporter (NBC) was blocked the astrocytes swelled by 8.3±0.7%, and 3) in the presence of an extracellular carbonic anhydrase (CA) inhibitor astrocytes swelled by 11.4±0.6%. Because a significant HCO_3−_ efflux is known to occur through the γ-amino-butyric acid (GABA) channel, we performed a series of experiments to determine if astrocytes were capable of HCO_3−_ mediated volume shrinkage with GABA channel activation. Astrocytes were found to shrink −7.7±0.5% of control in response to the GABA_A_ channel agonist muscimol. Astrocyte shrinkage from GABA_A_ channel activation was significantly decreased to −5.0±0.6% of control in the presence of the membrane-permeant CA inhibitor acetazolamide (ACTZ). These dynamic astrocyte volume changes may represent a previously unappreciated yet fundamental mechanism by which astrocytes regulate physiological brain functioning.

## Introduction

Neuronal firing of action potentials leads to the release of potassium ions (K^+^) into the extracellular space (ECS) of neural tissue and CO_2_ extrusion from the active neurons causes localized pH changes in the ECS. Astrocytes play an essential role in maintaining optimal physiological brain functioning by tightly regulating the concentrations of K^+^ and pH in the ECS, as alterations can affect a wide range of cellular processes that include neuronal excitability, synaptic transmission, metabolism, and the activity of many enzymes, channels, and transporters [1]–[5]. Astrocytes undergo dramatic volume changes as a result of the ionic dysregulation that occurs during pathological brain states such as ischemia, trauma and epilepsy [6]–[9]. Previous experimental work have nicely described astrocyte volume changes in conjunction with extracellular K^+^ buffering [10]–[15] however, these studies were conducted using large concentrations of K^+^, well outside physiological range. As a result, the currently available literature reflects only what might be occurring during pathological states [16], and whether volume changes may be an intrinsic phenomenon of astrocytes that occurs during physiological brain functioning is not known.

Under physiological conditions even moderate amounts of neuronal activity can lead to a build up of extracellular K^+^. A single action potential can raise the [K^+^]_o_ by as much as 1 mM [17] and repetitive stimulation of a neuronal fibre pathway has been reported to raise the [K^+^]_o_ to 12 mM [4], [18]. As well, astrocytes rapidly undergo an intracellular alkalinization in response to modest increases in [K^+^]_o_ [2], [19]. This alkalinization is dependent on the uptake of the bicarbonate ion (HCO_3−_), and can be blocked with the removal of extracellular HCO_3−_, or by inhibition of the main astrocyte HCO_3−_ transporter, the sodium-bicarbonate cotransporter (NBC) [20], [21]. In addition, reversible changes in the size and diffusion properties of the ECS have also been shown to occur under physiological conditions, which have been documented in response to neuronal activity [6], [8], [22], [23]. Indirect measurements of cellular volume, that can be acquired from changes in either the optical or diffusion properties of brain tissue, have long been assumed to be due to astrocyte volume changes [6], [24]. However, the relationship between the intrinsic optical properties of tissue and changes in cellular swelling has been disputed in live preparations [25].

In the present study we performed two-photon imaging in the brain slice preparation to directly examine real-time astrocyte volume changes in the stratum radiatum of the hippocampus CA1 region. Astrocytes were found to undergo robust swelling in response to a physiological increase in [K^+^]_o_, and to shrink in response to GABA_A_ receptor activation. Both the astrocyte swelling and shrinking were mediated by the flux of HCO_3−_. These dynamic astrocyte volume changes may represent a previously unappreciated yet fundamental mechanism by which astrocytes regulate physiological brain functioning.

## Materials and Methods

### Hippocampal Slice Preparation

This work was approved by the University of Saskatchewan’s Animal Research Ethics Board, and adhered to the Canadian Council on Animal Care guidelines for humane animal use. Male Sprague Dawley rats, aged 17–22 days postnatal were anesthetized with halothane, decapitated, and the brains rapidly removed. Horizontal hippocampal slices (400 µm thick) were cut using a vibrating microtome (*VT1200, Leica Microsystems, Nussloch, Germany*) in ice-cold (0–4°C) sucrose cutting solution containing (in mM): 87 NaCl, 75 Sucrose, 2.5 KCl, 25 NaHCO_3_, 10 Glucose, 7 MgCl_2_, and 0.5 CaCl_2_ oxygenated with 95% O_2_ - 5% CO_2_. Slices were incubated in artificial cerebrospinal fluid (ACSF) containing (in mM): 125 NaCl, 2.5 KCl, 25 NaHCO_3_, 10 Glucose, 2 MgCl_2_, 1.25 NaH_2_PO_4_, and 2 CaCl_2_ at 32°C for 30 minutes and then room temperature until use. Following a minimum of 30 minutes equilibration time at room temperature, slices were transferred to a submerged recording chamber and constantly perfused (∼2 mL/min) with oxygenated ACSF (pH 7.4; osmolality, 295–300 mOsm) maintained at 25±1°C using an inline solution heater (*Warner Instruments*). For HCO_3−_ free experiments, slices were transferred into 4-(2-hydroxyethyl)-1-piperazineethanesulfonic acid (HEPES) buffered ASCF containing (in mM): 130 NaCl, 2.5 KCl, 25 HEPES, 10 Glucose, 2 MgCl_2_, 1.25 NaH_2_PO_4_, and 2 CaCl_2_, bubbled with 100% O_2_ and buffered to a pH of 7.4 with NaOH. High K^+^ solutions were prepared by adding 3 mM KCl and removing an equimolar amount of NaCl to HCO_3_^−^ and HEPES buffered recipes. To selectively label astrocytes, slices were incubated for 15 minutes in 25–100 µM SR101 [26] then perfused continuously with ACSF for 2 hours before imaging.

### Two-photon Imaging

We performed two-photon imaging using a custom modified Olympus BX51WIF upright research microscope interfaced with an Ultima – X–Y laser – scanning module (*Prairie Technologies Inc.)* directly coupled to a Mai Tai XF (Spectra Physics) mode-locked Ti: Sapphire laser source. Images were acquired using an Olympus 40X water immersion objective lens (LUMPLFL 40XW/IR-2). SR101 labeled astrocytes in the stratum radiatum of the CA1 region of the hippocampus were imaged deeper than a minimum of 50 µm below the surface of the slice. The SR101 fluorophore was excited at 835 nm and the resulting epifluorescence detected with a top-mounted low dark current (<10 nA) high sensitivity (>8500 A/lumen) external PMT detector (*Hamamatsu*) fitted with a red bandpass emission filter (607/45 nm). To image astrocyte volume changes, z-stack images (1 µm step) were acquired with an X-Y spatial resolution of 0.29 µm/pixel.

### Image Analysis

Z-stacks taken at each imaging time point throughout an experiment were compressed into single, two-dimensional maximum intensity projections, overlayed, aligned, and thresholded at the same level to remove image background and leave only clearly defined astrocyte somas and the initial primary processes. To compensate for the poor axial resolution inherent with two-photon microscopy (∼2 µm), high-resolution area measurements were taken from the two-dimensional maximum intensity projections of the image stacks. To eliminate measuring bias that can be created by manually tracing or outlining astrocytes, individual astrocytes within each image projection were cropped and the areas calculated in ImageJ software. The percent changes in the areas of the astrocytes at different experimental time points were adequate to determine relative astrocyte volume changes, assuming that astrocyte soma volume is changing uniformly in all directions, but underestimate actual volume changes [9]. Prior to any analysis, the health of each hippocampal slice was determined based on astrocyte morphology and the quality of the SR101 dye loading. All trials where the cells were not clearly defined or where the dye intensity was not stable for the duration of the experiment were discarded.

### Drug Application

All chemicals were bath applied. The membrane impermeant CA inhibitor 1-(4-sulfamoylphenylethyl)-2,4,6-trimethyl-pyridinium perchlorate (STPP)(100 µM), received as a generous gift from Dr. Claudio Supuran (University of Florence, Italy), 4,4’-diisothiocyanostilbene-2,2’-disulfonic acid (DIDS)(*Sigma*)(500 µM), and Acetazolamide (*Sigma*)(100 µM) were made up in DMSO and applied for a minimum of 30 minutes before imaging. The concentration of DMSO in the final solution never exceeded 0.1%. Muscimol (*Tocris Bioscience*) (20 µM) was dissolved in ddH_2_O.

### Statistics

Significant differences between population means were assessed using either a Student’s t-test or a one-way ANOVA followed by a Bonferroni post-hoc analysis, where appropriate, with a confidence level of p<0.05. Results are presented as mean ± standard error of the mean (SEM). All statistical analyses were performed using the software program PASW Statistics version 18.0.

## Results

### Astrocyte Volume Remains Stable during Long-term Imaging

**Figure 1:**
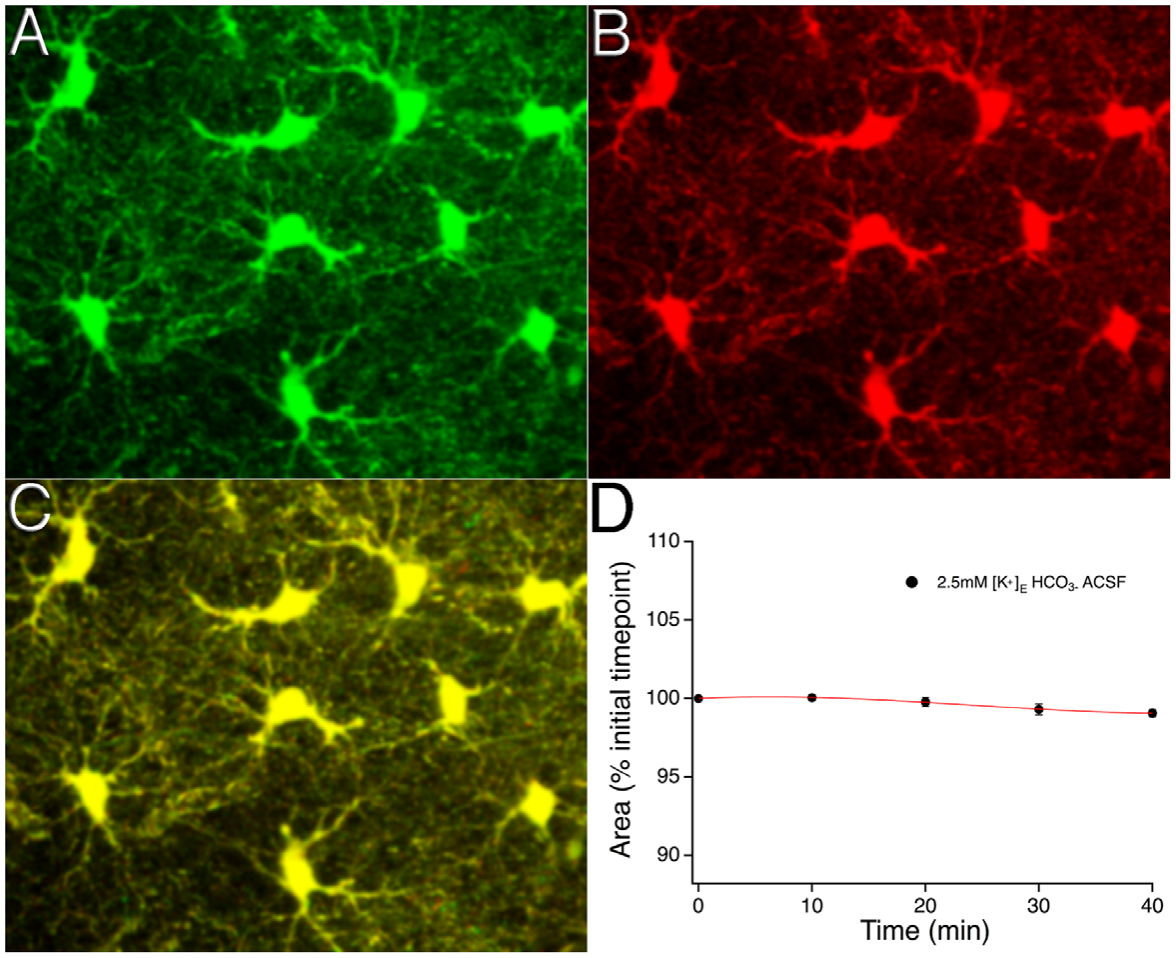
Reliable quantification of astrocyte area during long-term imaging. Two-photon z-stack projection images of SR101 labeled astrocytes in the CA1 stratum radiatum region of the hippocampus in control (2.5 mM [K^+^]_o_) ACSF at time zero (**A**) and after a 40-minute experimental period (**B**). The merged image (**C**) shows the extent of astrocyte overlap within the field of view at the two experimental timepoints. (**D**) The time course of the astrocyte area changes over a 40-minute experimental period; error bars at each 10-minute image acquisition time point denote SEM.

Two-photon microscopy was used to image SR101 labeled astrocytes in the CA1 stratum radiatum region of the hippocampus to determine if stable measurements of astrocyte area could be achieved during long-term imaging experiments. The average astrocyte area (soma + primary processes) was found to be 113.0±2.1 µm^2^ (n = 314 from 57 slices). Astrocyte area changed ∼1% over 40 minute experimental time periods in control ACSF during which z-stack image series were taken every 10 minutes (percentage change: −0.9±0.2%, n = 24 astrocytes from 4 brain slices) (figure 1).

### Astrocyte Swelling in Response to Increased [K^+^]_o_ within Physiological Range is Mediated by HCO_3_^−^ Influx

**Figure 2:**
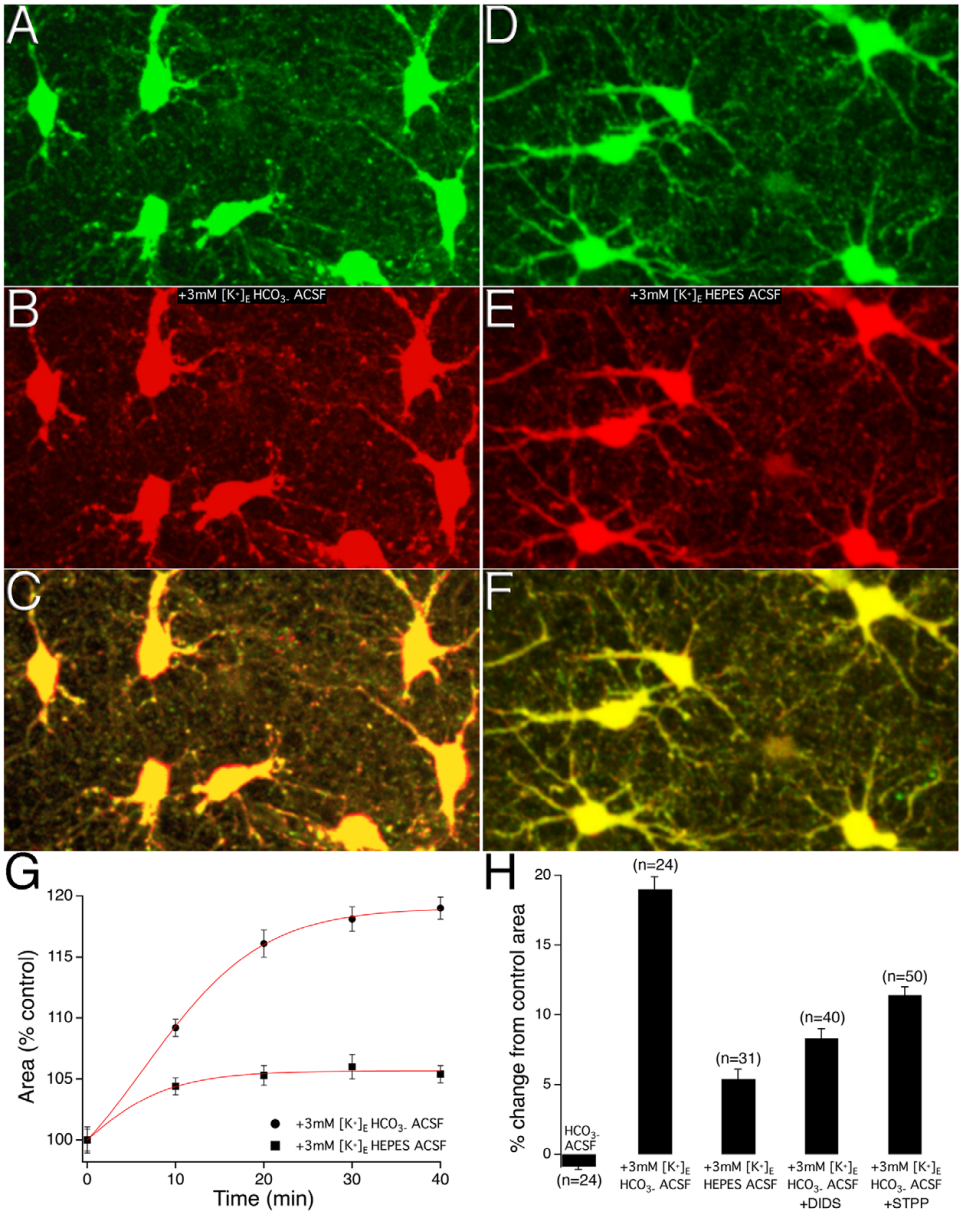
HCO_3_^−^ influx mediates increased [K^+^]o –induced astrocyte swelling. **A**) Images show astrocytes in 2.5 mM [K^+^]_o_ ACSF at time zero and after maximal swelling has occurred in 5.5 mM [K^+^]_o_ at the 40-minute time point (**B**). **C**) Merged image shows clearly defined regions of astrocyte area changes (*red*). **D**) Images of astrocytes in 2.5 mM [K^+^]_o_ HCO_3_^−^ free (HEPES buffered) ACSF at time zero and after maximal swelling has occurred with the addition of 3 mM [K^+^]_o_ at the 40-minute time point (**E**). **F**) Merged image does not display clearly defined regions of astrocyte area changes. **G**) The time courses of the +3 mM [K^+^]_o_ –induced astrocyte area changes under conditions of HCO_3_^−^ ACSF and HCO_3_^−^ free (HEPES buffered) ACSF; error bars at each 10-minute image acquisition time point denote SEM. **H**) Bar graph summary of the maximum percent changes in astrocyte area in conditions of 2.5 mM [K^+^]_o_ HCO_3_^−^ ACSF, +3 mM [K^+^]_o_ HCO_3_^−^ ACSF, +3 mM [K^+^]_o_ HCO_3_^−^ free ACSF, +3 mM [K^+^]_o_ +DIDS, and +3 mM [K^+^]_o_ +STPP. Error bars denote the SEM.

To determine whether astrocyte swelling might occur under non-pathological conditions, hippocampal slices were perfused with ACSF containing 5.5 mM [K^+^]_o_ from an initial control ACSF containing 2.5 mM [K^+^]_o_. Figure 2 shows images of astrocytes in 2.5 mM [K^+^]_o_ ACSF (A *green*) and after maximal swelling has occurred with the 3 mM increase of [K^+^]_o_ (B *red*). The overlayed image (figure 2C) clearly shows regions (red) around all astrocyte somas within the field of view indicating area increases with perfusion of ACSF containing 5.5 mM [K^+^]_o_ (movie S1). To establish the time-course of the +3 mM [K^+^]_o_ –induced astrocyte volume changes, a series of z-stack images occurring at 10 minute intervals were taken of the astrocytes. Astrocytes were observed to swell by 9.3±0.7% at the 10minute time point, 16.1±1.1% at the 20 minute time point, 18.1±1.0% at the 30 minute time point, and by a maximal amount of 19.0±0.9% at the 40 minute time point; n = 24 astrocytes from 6 brain slices (figure 2G). The +3 mM [K^+^]_o_ –induced astrocyte volume changes were significant compared to those astrocytes imaged over the same 40 minute experimental time period while remaining in the 2.5 mM [K^+^]_o_ ACSF [t(46) = 22.094, p<0.05] (figure 2H).

To investigate a role for HCO_3_^−^ in the +3 mM [K^+^]_o_ –induced astrocyte swelling, we employed three different conditions expected to reduce HCO_3_^−^ influx into astrocytes. In a first series of experiments the level of extracellular HCO_3_^−^ was controlled by incubating hippocampal slices in HCO_3_^−^ free, 4-(2-hydroxyethyl)-1-piperazineethanesulfonic acid (HEPES) buffered ACSF. HCO_3_^−^ free ACSF significantly decreased the +3 mM [K^+^]_o_ –induced astrocyte swelling compared to the swelling observed under HCO_3_^−^ buffered ACSF, conditions; 5.4±0.7%, n = 31 astrocytes from 7 slices, p<0.05 (figure 2D–H). In a second series of experiments, the level of extracellular HCO_3_^−^ was reduced using a membrane impermeant extracellular carbonic anhydrase (CA) inhibitor to decrease the production of extracellular HCO_3_^−^. The membrane impermeant CA inhibitor 1-(4-sulfamoylphenylethyl)-2,4,6-trimethyl-pyridinium perchlorate (STPP) (100 µM) was used to selectively inhibit the formation HCO_3_^−^ in the extracellular solution. +3 mM [K^+^]_o_ –induced astrocyte swelling was significantly reduced in the presence of STPP; 11.4±0.6%, n = 50 cells from 7 slices, p<0.05 (figure 2H). A third set of experiments was performed to directly block HCO_3_^−^ influx through the sodium-bicarbonate cotransporter (NBC) cotransporter. Inhibition of NBC activity with the NBC inhibitor DIDS (500 µM) significantly decreased the +3 mM [K^+^]_o_ –induced astrocyte swelling; 8.3± 0.7%, n = 40 astrocyte from 6 slices, p<0.05 (figure 2H).

### HCO_3_^−^ Efflux Plays a Role in GABA Channel Mediated Astrocyte Shrinkage

**Figure 3:**
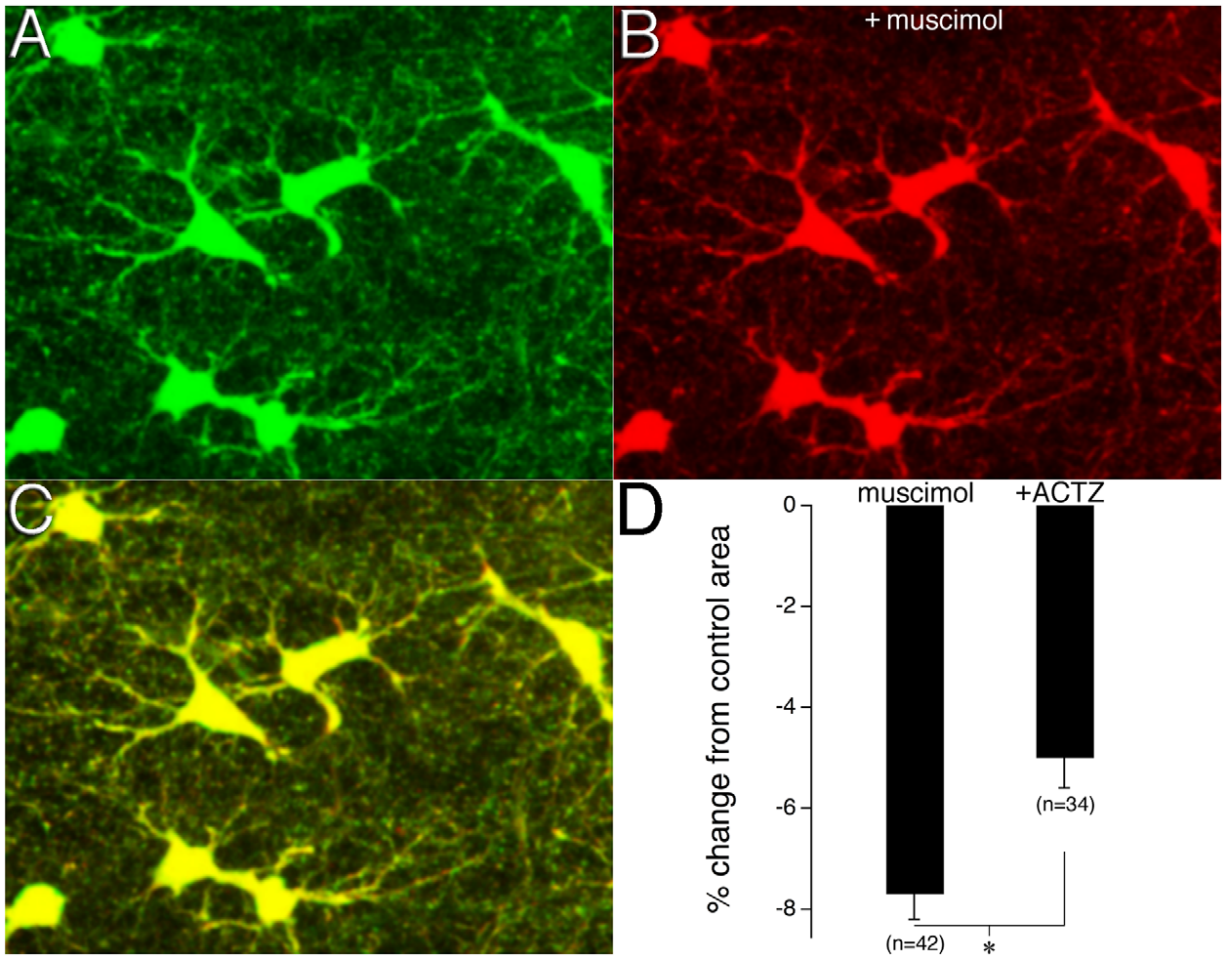
GABA channel HCO_3_^−^ efflux plays a role in astrocyte shrinkage. **A**) Images show astrocytes in control ACSF and after the addition of the selective GABA_A_ agonist muscimol (**B**). **C**) Merged image shows clearly defined regions of astrocyte area changes (*green*). **D**) Bar graph shows the percent changes in astrocyte area induced by muscimol and by muscimol in the presence of ACTZ. Error bars denote the SEM; asterisk indicates a significance difference between muscimol, and muscimol +ACTZ groups.

The selective GABA_A_ agonist muscimol was used determine whether GABA_A_ channel activation caused astrocyte volume changes. Muscimol (20 µM) was found to cause significant astrocyte shrinking; −7.7± 0.5% of control, n = 42 astrocyte from 8 slices, p<0.05) (figure 3 and movie S2). To determine whether HCO_3_^−^ played a role in GABA_A_ induced astrocyte shrinkage via efflux through the GABA_A_ channel, the membrane-permeant CA inhibitor acetazolamide (ACTZ) (100 µM) was used to block the formation of intracellular HCO_3_^−^ during muscimol applications. ACTZ significantly decreased the amount of GABA_A_ channel mediated astrocyte shrinkage by muscimol; −5.0±0.6% of control, n = 34 astrocytes from 7 slices, p<0.05 (figure 3D). A Student’s t-test confirmed that there was no significant difference between the shrinkage observed in control astrocytes that were incubated in 2.5 mM [K^+^]_o_ for 40 minutes and astrocytes that were incubated with ACSF containing ACTZ [t(4.57) = 1.45, p = 0.212]. A one-way ANOVA confirmed significance between the control, muscimol, and muscimol with ACTZ groups (*F*(2,97) = 46.28, p<0.05).

## Discussion

In the present study, two-photon imaging was used to directly examine real-time astrocyte volume changes in the stratum radiatum of the hippocampus CA1 region. Astrocytes were found to swell in response to a 3 mM increase in [K^+^]_o_, a concentration that is well within physiological range, and to shrink in response to GABA_A_ receptor activation. These dynamic volume changes were regulated in a HCO_3_^−^ dependent manner.

### High-resolution Imaging and Quantification of Astrocyte Volume Changes

To the best of our knowledge this is the first study to report on astrocyte volume changes in the brain slice preparation that resulted from non-pathological induction methods. We were able to achieve high image resolution and stable measurements of astrocyte area during long-term imaging experiments and determined that astrocyte area changed less than 1% over 40 minutes in control ACSF, while a modest +3 mM increase in [K^+^]_o_ caused a 19% increase and GABA_A_ channel activation an 8% decrease for the same time period. A number of recent elegant studies have used comparable high-resolution imaging approaches to investigate dynamic astrocyte volume changes in the brain slice preparation, and thus offer the opportunity for quantitative comparisons to be made between the magnitude of the astrocyte volume changes we report here and those that resulted from pathological conditions, such as osmotic stress, ischemia, and cortical spreading depression (SD) events [9], [27]–[31]. Zhou *et al*. (2010) induced spreading depression in the brain slice preparation by perfusing ACSF containing a +40 mM increase in [K^+^]_o_ and found that astrocytes swelled 40% [31]. Importantly, their study was able to show that astrocytes did not exhibit volume changes associated with SD but only displayed passive swelling in response to high [K^+^]_o_, which suggests that astrocytes are not the primary contributor to SD propagation. As in the Zhou *et al*. (2010) study, Risher *et al*. (2009) found that brain slices perfused with ACSF containing high [K^+^]_o_ (+26 mM) also caused a doubling in the magnitude of astrocyte swelling compared to our findings, showing that astrocytes swelled 38.9% [9]. Interestingly, the magnitude of the +26 mM [K^+^]_o_ -induced astrocyte swelling was greater than the astrocyte swelling that resulted from −40mOsm osmotic challenge (17%) and oxygen/glucose deprivation (29%) [9]. In two different studies, Benesova *et al*. found that astrocytes swelled to a maximum of 40% [27] and 36% [28] of control conditions with oxygen/glucose deprivation in the brain slice preparation. A 40% increase in the area of astrocyte somas was also observed in slices perfused with hypoosmotic solution (∼65% of control osmolality) [29]. Two photon laser scanning microscopy has also been used *in vivo* to examine osmotic- and ischemia-induced volume changes in astrocytes. Astrocyte somas were shown to swell by 21% as a result of hypoosmotic stress induced by IP distilled water injection and 33% from induced global ischemia induced from cardiac arrest [9]. As well, using a photothrombotic occlusion model, Risher *et al*. (2012) showed that astrocyte somas in the metabolically compromised ischemic penumbra-like area swelled by 31% as a result from the initial induced spreading depolarizations and by 30% as a result of spreading depolarization induced by a transient bilateral common carotid artery occlusion model of global ischemia [30].

The robust magnitude of the astrocyte volume changes found in the present study that occurred from the modest induction methods compared to the studies above, likely result from the experimental paradigm used here; that is, the +3 mM [K^+^]_o_ and muscimol once applied, were perfused for the remainder of imaging experiments. While this model allowed us to very precisely quantify astrocyte volume changes and unmask a previously underestimated contribution of HCO_3_^−^ flux, and certainly does comment on the astonishing propensity for astrocytes to change their volumes, it in no way predicts the likely magnitude of astrocyte volume changes that are to occur during normal physiological brain functioning. Indeed, currently available *in vivo* data indicates that [K^+^]_o_ changes in a fluctuating manner in the ECS and that modest astrocyte volume changes may occur in this manner as well under physiological brain functioning, such as during slow (∼1 Hz) sleep oscillations [32].

### Astrocyte Volume Changes are Dependent on HCO_3_^−^ Flux

For normal neuronal functioning to be maintained, the local changes in [K^+^]_o_ that occur as a result of neural activity must be tightly regulated. Astrocytes are thought to play a key role in this regulation by taking up excess K^+^ from the ECS [4], [33]. In response to increases in [K^+^]_o_ astrocytes are known to rapidly undergo an intracellular alkalinization [2], [19] however, the involvement of HCO_3_^−^ in [K^+^]_o_ -induced astrocyte swelling under physiological conditions was only recently speculated [34]. In the present study, we found that astrocytes undergo robust swelling in response to a physiological increase in [K^+^]_o_ and that the removal of HCO_3_^−^ from the extracellular solution, inhibition of the extracellular CA activity, and inhibition of the NBC activity all resulted in significant decreases in the magnitude of the [K^+^]_o_ -induced swelling.

Astrocyte GABA_A_ receptor activation has been shown to induce an acidification of the astrocyte cytoplasm and an alkalinization of the ECS [35], [36]. Because this acidification is blocked by the removal of HCO_3_^−^ from the system or with a CA inhibitor to block the formation of HCO_3_^−^ it is believed to occur as a result of net HCO_3_^−^ efflux through the GABA_A_ channels [1], [36]–[39]. Astrocytes allow both HCO_3_^−^ and chloride (Cl^-^) efflux [40] with a suspected HCO_3_^−^ to Cl^-^ permeability ratio of 0.2 [41], [42] and in a recent study it was elegantly shown that in hippocampal mature astrocytes GABA_A_ receptor activation mediated HCO_3_^−^ efflux depolarized the astrocyte membrane potential [43]. Here we find that astrocytes shrink with GABA_A_ channel activation and that this shrinkage was significantly decreased with inhibition of CA. Taken together, these results provide evidence for a HCO_3_^−^ dependent mechanism in astrocyte volume changes under non-pathological conditions. It will be important to confirm our findings with direct HCO_3_^−^ flux measurements as HCO_3_^−^ selective optical probes become available [44].

Our findings support a mechanism for K^+^ induced astrocyte swelling that was first proposed by Nagelhus and colleagues (2004) [20]. This proposal suggested that increases in the [K^+^]_o_ as a result of neuronal activity causes a net driving force for K^+^ influx into astrocytes, while at the same time causes a depolarization of the astrocyte membrane. This depolarization is believed to activate the uptake of Na^+^ and HCO_3_^−^ through the NBC, which is highly expressed in astrocytes [45]–[47]. The resulting accumulation of K^+^, Na^+^, and HCO_3_^−^ in the astrocyte cytoplasm would then generate an osmotic gradient and drive water influx through aquaporin 4 water channels (AQP4) that are highly expressed in astrocytes [20], [48]. Interestingly, this mechanism would serve to clear the ionic bi-product, K^+^, and the metabolic bi-product, CO_2_, of neuronal activity from the ECS at the same time. Recent work from the Nagelhus group suggests that activity-dependent astrocyte water uptake may be partly offset by an AQP4-mediated water efflux that may serve to moderate the activity-dependent astrocyte volume changes and offset the resulting ECS shrinkage [49].

Removal of HCO_3_^−^ from the extracellular solution, inhibition of the extracellular CA activity, and inhibition of the NBC activity all resulted in significant decreases in the magnitude of the [K^+^]_o_ -induced swelling, but each of the individual conditions did not fully prevent the astrocyte swelling. A wide variety of other transporters are known to contribute to the regulation of astrocyte volume. These include the sodium-potassium chloride cotransporter (NKCC), the K^+^-Cl^-^ cotransporter (KCC), the Cl^–^HCO_3_^−^ anion exchanger (AE), the Na^+^ dependent Cl^–^HCO_3_^−^ exchanger (NDCBE) and the Na^+^-H^+^ exchangers (NHE) (reviewed in [50]; [20], [34]). It is likely that one or more of these transporters contributes to the +3 mM [K^+^]_o_ –induced astrocyte swelling found in the present study. The cotransporter that has been most widely implicated in K^+^ induced astrocyte swelling is the NKCC1 form of the NKCC cotransporter. The NKCC1 actively transports one each of K^+^ and Na^+^ and two Cl^-^ ions into the astrocyte cytoplasm, generating an osmotic gradient capable of driving water influx [51]. Inhibition of NKCC1 has been shown to reduce prominent astrocyte swelling that was induced by increases in the [K^+^]_o_ of up to 50 mM or more [10], [11], [13]. In an effort to provide a clearer explanation of the mechanisms underlying the phenomenon of extracellular space (ECS) shrinkage due to astrocyte swelling, Otsby and colleagues (2009) performed a mathematical modeling study and found that the activity of either NKCC1 or NBC, in addition to passive ion transport mechanisms, appears to be necessary to account for astrocyte swelling on a scale that would produce shrinkage of the ECS [34].

### Implications for Brain Functioning

Because astrocyte swelling involves the removal of water from the ECS, the concentration of local solutes, such as ions and signaling molecules, may be increased [16]. Since extrasynaptic volume transmission constitutes a major signaling mechanism between brain cells [52]–[54], any changes in the diffusion properties of the ECS have the potential to greatly affect brain cell signaling [16]. Because astrocytes possess large numbers of highly branched processes, they constitute a major barrier to diffusion in the brain [54], [55]. Changes in the volume of astrocytes may have a significant effect on the ability of substances to diffuse within the ECS [13]. In this study measurements were restricted to the astrocyte somas and initial primary processes to avoid quantification error that might occur from the finer processes, given the limits of optical resolution or as a result of dye leakage. Thus, volume changes that may be occurring in a significant portion of each astrocyte were not considered. However, given that the AQP4 water channels colocalize with inwardly rectifying K^+^ channels and expression is much higher in the finer processes that come in contact with glutamatergic synapses [20], [56], [57] than in the soma or primary processes, it is not unreasonable to speculate on the functional role of astrocyte volume changes that may be occurring as well in the finer processes. The intimate anatomical relationship between fine astrocyte processes and synapses in the brain facilitates the involvement of astrocytes in the regulation of synaptic plasticity [58]. The astrocyte fine processes contain high numbers of glutamate transporters [59], [60] that are essential for glutamate uptake from the synapse and the maintenance of low extracellular glutamate concentrations [61]. Upon release from an active excitatory synapse, glutamate can diffuse out of the synaptic cleft, or ‘spillover’ to stimulate extrasynaptic receptors and neighbouring synapses [62], [63]. In the hippocampus, glutamate spillover, and therefore the activation of extrasynaptic glutamate receptors, is limited by the activity of glutamate transporters on the surrounding astrocytes [62], [64]. Due to their involvement in glutamate uptake, astrocytes may be directly involved in the induction of one form of synaptic plasticity, long-term depression (LTD), which appears to occur when glutamate spillover from the synapse activates extrasynaptic NMDA receptors [63], [65]. The induction of LTD is facilitated when astrocyte glutamate uptake is blocked to allow for a greater amount of glutamate spillover from the synapse [63], [65].

A decreased ability of astrocytes to clear glutamate from around a synapse may occur if the astrocyte shrinks thereby increasing in the distance between the astrocyte processes and the synapse. The effect that distance between astrocyte processes and the synapse has on synaptic transmission has previously been demonstrated in the hypothalamus [55]. In the hypothalamus, reversible retraction of astrocyte processes occurs during times of lactation and dehydration [58]. As the processes retract further from the synapse, glutamate clearance from the ECS is delayed and the efficacy of surrounding synapses can be decreased [55], [58], [66]. The volume changes that we find here occur over a much shorter time period, and on a much smaller scale, than astrocyte process retraction in the hypothalamus. However, the experiments in the hypothalamus clearly demonstrate the importance that the proximity of astrocyte processes to the synapse plays in their ability to regulate synaptic function. Astrocyte swelling in response to extracellular K^+^, or shrinking in response to GABA_A_ receptor activation, is likely not restricted to the soma, but instead may extend to the furthest reaches of the astrocyte processes. In this manner, the volume changes observed in this study may play an integral role in the regulation of synaptic transmission over a much shorter time span than what has previously been observed in the hypothalamus.

Finally, in an attempt to counteract swelling and to recover normal volume, astrocytes initiate volume regulatory mechanisms by modifying their concentration of osmotically active solutes through a process termed regulatory volume decrease (RVD). Volume-regulated anion channels (VRACs) are activated by increased cell volume and are an important conduit through which glutamate release from astrocytes occurs during pathological brain states [67]–[69]. Given the dynamic 3 mM [K^+^]_o_ –induced astrocyte volume changes seen in the current study, the possibility exists that VRAC mediated glutamate release may occur as a result of astrocytes swelling during modest physiological challenges as well and during normal patterns of neuronal activity in the healthy brain. Indeed, Mongin and Kimelberg (2005) used moderate astrocyte swelling to test for VRAC activity under conditions resembling those to which astrocytes may be exposed to upon physiological stimulation and elegantly demonstrated Ca^2+^-dependent VRAC glutamate release [70]. Their findings suggest this mechanism might have functional significance for physiological functioning in the brain. Glutamate released in this manner may substantially impact the extent of neural communication, because even modest increases in extracellular glutamate concentrations are capable of altering neuronal excitability, synaptic transmission, and activity-related synaptic plasticity.

### Conclusion

The results of this study demonstrate the existence of dynamic astrocyte volume changes that may be an intrinsic phenomenon that occur under physiological conditions. These volume changes may play a fundamental role in the control of extracellular brain signaling and in the regulation of synaptic plasticity.

## Supporting Information

Movie S1 **This file contains a movie showing astrocyte swelling and recovery in a series of two-photon z-stack projection images of SR101 labeled astrocytes in the CA1 stratum radiatum region of the hippocampus.** Images were acquired every 10 minutes over the course of 100 minutes. At time zero the slice was switched from 2.5 mM [K^+^]_o_ ACSF to 5.5 mM [K^+^]_o_ ACSF. After the 40-minute time point the perfusate was switched back to 2.5 mM [K^+^]_o_.
(MOV)

Movie S2 **This file contains a movie showing astrocyte shrinking from the selective GABA_A_ agonist muscimol.** At time zero muscimol was added to the ACSF and images were acquired every 10 minutes for 40 minutes.
(MOV)
